# Cerebral white matter structure is associated with DSM-5 schizophrenia symptom dimensions

**DOI:** 10.1016/j.nicl.2016.06.013

**Published:** 2016-06-16

**Authors:** Petra V. Viher, Katharina Stegmayer, Stéphanie Giezendanner, Andrea Federspiel, Stephan Bohlhalter, Tim Vanbellingen, Roland Wiest, Werner Strik, Sebastian Walther

**Affiliations:** a Translational Research Center, University Hospital of Psychiatry, Bern, Switzerland; b Department of Clinical Research, Inselspital, Bern, Switzerland; c Neurology and Neurorehabilitation Center, Luzerner Kantonsspital, Luzern, Switzerland; d Support Center of Advanced Neuroimaging (SCAN), University Institute of Diagnostic and Interventional Neuroradiology, Inselspital, Bern, Switzerland

**Keywords:** Diffusion tensor imaging, Tract-Based Spatial Statistics, Neurobiological correlates, Negative syndrome, Motor abnormalities

## Abstract

Diffusion tensor imaging (DTI) studies have provided evidence of widespread white matter (WM) abnormalities in schizophrenia. Although these abnormalities appear clinically significant, the relationship to specific clinical symptoms is limited and heterogeneous. This study examined the association between WM microstructure and the severity of the five main DSM-5 schizophrenia symptom dimensions. DTI was measured in forty patients with schizophrenia spectrum disorders. Using Tract-Based Spatial Statistics controlling for age, gender and antipsychotic dosage, our analyses revealed significant negative relationships between WM microstructure and two DSM-5 symptom dimensions: Whereas abnormal psychomotor behavior was particularly related to WM of motor tracts, negative symptoms were associated with WM microstructure of the prefrontal and right temporal lobes. However, we found no associations between WM microstructure and delusions, hallucinations or disorganized speech. These data highlight the relevance of characteristic WM disconnectivity patterns as markers for negative symptoms and abnormal psychomotor behavior in schizophrenia and provide evidence for relevant associations between brain structure and aberrant behavior.

## Highlights

• DTI study of brain-behavior associations of the new DSM-5 schizophrenia dimensions.
• The severity of the DSM-5 abnormal psychomotor behavior dimension is related to white matter microstructure in motor tracts.
• Associations of the DSM-5 negative symptom dimension with white matter microstructure are found in prefrontal and temporal clusters.
• Characteristic patterns of white matter microstructure argue for relevant associations between brain structure and aberrant behavior in schizophrenia.

## 1 Introduction

Schizophrenia is characterized by heterogeneous symptom patterns. This heterogeneity has long been explained in terms of clinical subtypes, as in the fourth edition of the Diagnostic and Statistical Manual of Mental Disorders (DSM-IV) (Tandon et al., 2013). Because these subtypes lack stability and biological correlates, they were eliminated and replaced by psychopathological dimensions in the DSM-5 (Barch et al., 2013, Peralta and Cuesta, 2001). The main symptom dimensions of schizophrenia in the DSM-5 are delusions, hallucinations, disorganized speech, abnormal psychomotor behavior, and negative symptoms. The DSM-5 schizophrenia dimensions allow describing the heterogeneity of symptoms in a more valid, reliable and clinically useful way (Tandon et al., 2013).

Brain white matter (WM) abnormalities have been reported as one of the central hallmarks in schizophrenia; thought to contribute to the pathophysiology of the disorder (Davis et al., 2003). Diffusion tensor imaging (DTI) is a non-invasive Magnetic Resonance Imaging (MRI) method that allows the investigation of WM microstructure by quantifying the degree and direction of water diffusion (Basser, 1995, Mori et al., 2005). A number of studies provide evidence of WM abnormalities in schizophrenia, predominantly in the prefrontal and temporal lobe, using most often fractional anisotropy (FA) as an indicator of the integrity of WM (Federspiel et al., 2006, Kubicki et al., 2007). Altered white matter microstructure has been reported across the course of schizophrenia, in subjects at risk for psychosis and in healthy first-degree relatives of schizophrenia patients, suggesting that these alterations are associated both with the biological risk of schizophrenia and with symptom progression (Federspiel et al., 2006, Fitzsimmons et al., 2013, Ohtani et al., 2015).

Several studies have focused on the link between brain WM abnormalities and schizophrenia symptom dimensions. For example, conflicting results were reported for WM associations with the classical positive/negative symptom dimensions probably due to different patient groups (Asami et al., 2014, Cheung et al., 2011, Roalf et al., 2015, Zhang et al., 2016). For other symptom dimensions (e.g. disorganized speech, abnormal psychomotor behavior), only a few studies have investigated the association with aberrant WM integrity (Asami et al., 2013, Bai et al., 2009). In addition, cognitive impairment in schizophrenia was associated with WM microstructure in association fibers such as the right cingulum bundle, superior and inferior longitudinal fasciculi and inferior fronto-occipital fasciculus (Liu et al., 2013, Seitz et al., 2016, Zeng et al., 2016). Given the heterogeneity of current results for some symptom dimensions and a lack of studies for others, the question remains whether different dimensions of schizophrenia have specific brain WM correlates. Applying the dimensional approach of the DSM-5 to schizophrenia psychopathology may further help finding neural underpinnings of aberrant behavior (Heckers et al., 2013). Therefore, we aimed to investigate for the first time the association between WM microstructure and all of the five main DSM-5 dimensions in one group of patients with schizophrenia spectrum disorders. We hypothesized in some of the five dimensions an association between symptom severity and WM alteration, particularly in the dimension of abnormal psychomotor behavior, as aberrant motor behavior was consistently linked to WM abnormalities in schizophrenia before (Bracht et al., 2013, Walther et al., 2011).

## 2 Material and methods

### 2.1 Participants

Forty patients (25 men, 15 women) with schizophrenia (77.5%), schizophreniform (17.5%) or schizoaffective disorder (5%) were included in the study. All patients were recruited from the inpatient and outpatient departments of the University Hospital of Psychiatry Bern, Switzerland. They were right-handed as determined by the Edinburgh handedness inventory (Oldfield, 1971).

Inclusion criteria were diagnoses of schizophrenia, schizoaffective disorder or schizophreniform disorder according to the Diagnostic and Statistical Manual of Mental Disorders, fifth edition. Exclusion criteria were any substance-related addiction other than nicotine, a past or current medical or neurological condition associated with either impaired or aberrant movement or WM abnormalities (e.g. stroke, multiple sclerosis), histories of head trauma with loss of consciousness or electroconvulsive treatment and specific exclusion criteria for MRI scans (e.g. metallic implants, pregnancy and claustrophobia).

The severity of each core domain of the DSM-5 schizophrenia symptom dimensions was rated on a five-point scale ranging from 0 (not present) to 4 (present and severe) (Barch et al., 2013). Dimensional ratings of the severity in our group were: delusions (*M* = 2.18 ± 1.26), hallucinations (*M* = 1.3 ± 1.51), disorganized speech (*M* = 1.63 ± 1.37), abnormal psychomotor behavior (*M* = 1.53 ± 1.52) and negative symptoms (*M* = 2.2 ± 1.09). Correlations between the five symptom dimensions were calculated using Spearman rank correlations. Additional assessments included the Mini International Neuropsychiatric Interview (MINI) (Sheehan et al., 1998) and the Comprehensive Assessment of Symptoms and History (CASH) (Andreasen et al., 1992) to establish diagnoses and the Positive and Negative Syndrome Scale (PANSS) (Kay et al., 1987) for schizophrenia psychopathology. All but three patients received antipsychotic pharmacotherapy. Chlorpromazine equivalent dosages (CPZ) were calculated to evaluate current antipsychotic exposure (Woods, 2003). The demographic and clinical characteristics of the participants are summarized in Table 1.

The protocol was approved by the local ethics committee (KEK-BE 025/13) and adhered to the Declaration of Helsinki. All participants received oral and written information on the planned study. The capacity of the patients to give informed consent was confirmed by their treating psychiatrist. All participants provided written informed consent.

### 2.2 MRI acquisition

Imaging was performed on a 3 T MRI scanner (Siemens Magnetom Trio; Siemens Medical Solutions, Erlangen, Germany) with a 12-channel radio frequency headcoil for signal reception. For DTI measurements, we used a spin echo planar imaging (EPI) sequence (59 slices, FOV = 256 × 256 mm^2^, sampled on a 128 × 128 matrix, slice thickness = 2 mm, gap between slices = 0 mm, resulting in 2 mm^3^ isotopic voxel resolution) and TR/TE = 8000/92 ms covering the whole brain (40 mT/m gradient, 6/8 partial Fourier, GRAPPA factor 2, bandwidth 1346 Hz/Px). Diffusion-weighted images (DWI) were positioned in the axial plane parallel to the AC-PC line and measured along 42 directions with a *b*-value = 1300 s/mm^2^. The sequence included 4 images without diffusion weighting (e.g. *b*-value = 0 s/mm^2^; the first and every subsequent 12th image). We used a balanced and rotationally invariant diffusion-encoding scheme over the unit sphere to generate the DTI data. Acquisition time was 6 min.

### 2.3 DTI processing

DTI analyses were processed using the FMRIB (Functional Magnetic Resonance Imaging of the Brain's diffusion toolbox) Software Library (FSL) (FSL, http://www.fmrib.ox.ac.uk/fsl), including the Tract-Based Spatial Statistics (TBSS) software (Smith et al., 2006, Smith et al., 2004). The images of each subject were first corrected for head movements and eddy currents (using “eddy-tool” of FSL). FA images were created by fitting a tensor model to the raw data (using “FDT”) and then a brain extraction tool was applied (using “BET-tool” of FSL) (Smith, 2002). All subjects' FA data were aligned to a 1 mm^3^ Montreal Neurological Institute (MNI) standard space. The alignment was performed applying FMRIB's Non-Linear Image Registration Tool (Andersson et al., 2007a, Andersson et al., 2007b), which uses a b-spline representation of the registration warp field (Rueckert et al., 1999). A mean FA image was prepared and thinned in order to create a mean FA skeleton for later group comparisons. To prevent the inclusion of non-skeletal voxels, we used a FA threshold of 0.2. Each subject's aligned FA data was then projected onto the skeleton. The resulting data was subjected to voxel-wise between subject statistics. Additionally to the FA images, other parameters of DTI, mean diffusivity (MD), radial diffusivity (RD) and axial diffusivity (AD) were calculated by fitting a tensor model to the data at each voxel. In order to use TBSS in MD, RD and AD images, the nonlinear warps and skeleton projection of the FA images were applied to MD, RD and AD images using the “non_fa” option.

### 2.4 Statistical analysis

Statistical analyses for WM microstructure were carried out with TBSS, which is based on a non-parametric approach using permutation test theory with a general linear model (GLM) design matrix (Smith et al., 2006). Age, gender and chlorpromazine equivalents were entered as covariates of no interest into the analyses. Within the GLM framework, we examined the association between each of the five symptom dimensions with FA, MD, RD and AD.

Skeletonised FA, MD, RD and AD were tested voxelwise for associations with DSM-5 dimensions using a randomise tool (Winkler et al., 2014) (5000 permutations) with a threshold-free cluster enhancement (TFCE) correction method (Smith and Nichols, 2009). A TFCE corrected *p*-value < 0.05 (FWE corrected) was considered statistically significant in all of the analyses. The resulting significant skeletal regions were located and labelled by mapping the corrected statistical map to the Johns Hopkins University (JHU)-ICBM-DTI-81 WM labels atlas and the JHU-WM tractography atlas in MNI space (Mazziotta et al., 2001, Mori et al., 2005). All clusters exceeding 50 voxels per WM region were displayed.

## 3 Results

The correlations between behavioral ratings of the DSM-5 dimensions are summarized in Table 2. In two out of five dimensions, i.e. abnormal psychomotor behavior and negative symptoms, the severity of symptoms was specifically associated with WM brain structure. In contrast, we found no positive or negative linear relationship between WM microstructure and delusions (*p* ≥ 0.074), hallucinations (*p* ≥ 0.247) and disorganized speech (*p* ≥ 0.14). FA differences between schizophrenia patients and matched controls are given in the supplemental material. In short, patients had reduced FA in bilateral clusters within the corpus callosum, anterior limb of the internal capsule, corona radiata, anterior and posterior thalamic radiation, corticospinal tract, as well as in the superior and inferior longitudinal fasciculi.

### 3.1 Linear relationship of white matter microstructure and motor dimension

**Fig. 1:**
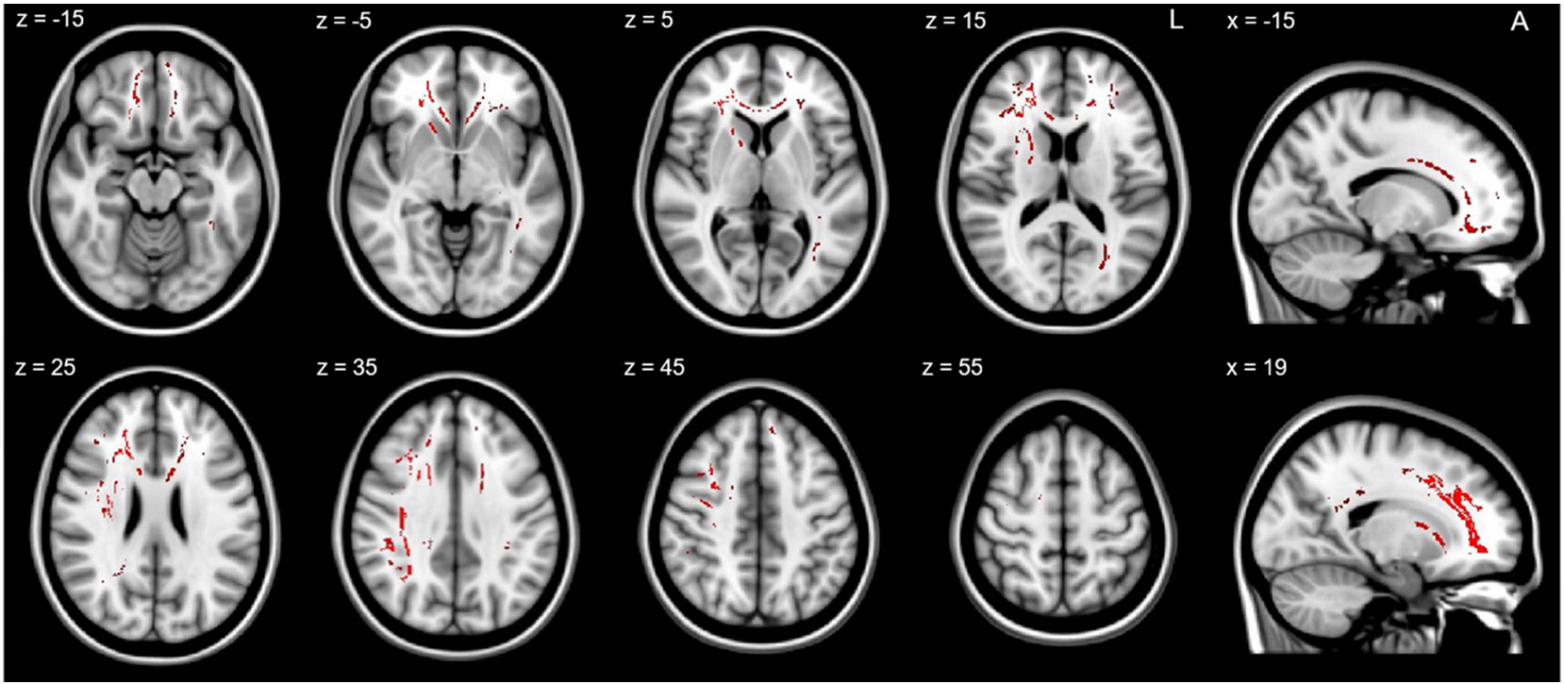
Relationship of white matter microstructure and abnormal psychomotor behavior. The TBSS image shows the negative linear relationship between the severity of the DSM-5 motor dimension and FA values within the areas indicated in red at *p* < 0.05, FWE corrected. Z and x indicate the coordinates of the image slices in mm. (For interpretation of the references to colour in this figure legend, the reader is referred to the web version of this article.)

The GLM applied in TBSS showed a significant negative linear relationship between the severity scores of the DSM-5 motor dimension and WM microstructure in predominantly motor tracts (see Fig. 1 and Table 3). Negative correlation was observed between FA and severity of the motor dimension in the superior and inferior longitudinal fasciculus, internal and external capsule and corpus callosum (*p* < 0.05, FWE corrected). Moreover, increased severity of the motor dimension was positively associated with increased RD (*p* < 0.05, FWE corrected) in similar regions of the frontal lobe (see Table 3). No associations with MD (*p* ≥ 0.101) and AD (*p* ≥ 0.386) were detected. All clusters with significant correlations of FA and the motor dimension were also found to have reduced FA in schizophrenia compared to healthy controls, except for the cluster within the left sagittal stratum and the right external capsule.

### 3.2 Linear relationship of white matter microstructure and negative symptom dimension

**Fig. 2:**
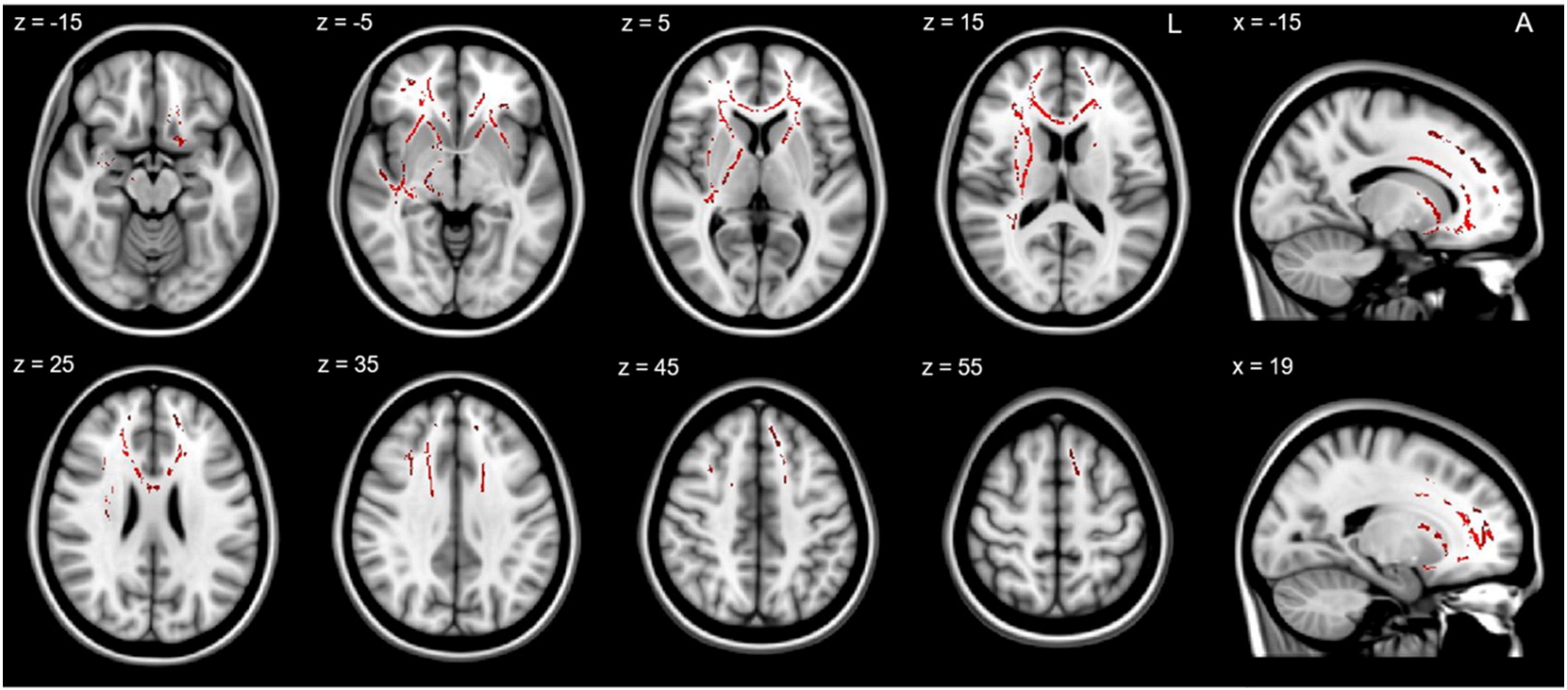
Relationship of white matter microstructure and negative symptoms. The TBSS image shows the negative linear relationship between the severity of the DSM-5 negative symptom dimension and FA values within the areas indicated in red at *p* < 0.05, FWE corrected. Z and x indicate the coordinates of the image slices in mm. (For interpretation of the references to colour in this figure legend, the reader is referred to the web version of this article.)

The GLM revealed a significant negative linear relationship between WM microstructure and the severity scores of the DSM-5 negative symptom dimension (see Fig. 2 and Table 4). Low FA values were associated with increased negative symptoms (*p* < 0.05, FWE corrected) in predominantly prefrontal clusters of the corona radiata, internal and external capsule and inferior fronto-occipital fasciculus. In addition, increased negative symptoms were associated with increased RD (*p* < 0.05, FWE corrected) in the corpus callosum and the anterior corona radiata (see Table 4). In contrast, MD (*p* ≥ 0.158) and AD (*p* ≥ 0.170) were not associated with the negative symptom dimension. Some of the clusters with significant association between FA and negative symptom severity were not detected in the group contrast (controls > patients, see supplemental material). These unique clusters include the cerebral peduncle, the right posterior limb of the internal capsule, sagittal stratum, the bilateral external capsule and uncinate fasciculus. However, bilateral clusters in the corpus callosum, corona radiata, anterior thalamic radiation and inferior fronto-occipital fasciculus overlap with those detected in the group comparison (see Supplemental material).

## 4 Discussion

This is the first study examining the associations of all five main DSM-5 schizophrenia symptom dimensions with WM microstructure in one group of patients with schizophrenia spectrum disorders. The results indicated characteristic patterns of WM microstructure linked to the severity of two DSM-5 schizophrenia symptom dimensions, i.e. abnormal psychomotor behavior and negative symptoms. WM integrity was associated with abnormal psychomotor behavior predominantly in motor tracts and with negative symptoms mainly in frontal regions.

Motor abnormalities are frequent in schizophrenia, but insufficiently studied with neuroimaging methods (Walther, 2015, Walther and Strik, 2012). Previous studies demonstrated an association of the activity level as a measure of motor behavior with WM regions underneath the supplemental motor area, precentral gyrus, cingulum and with connections between the primary motor cortex and subcortical structures and between the pre-SMA and other motor areas in patients with schizophrenia (Bracht et al., 2013, Walther et al., 2011). Furthermore, a DTI study in patients with schizophrenia reported correlations between dyskinesia severity and FA in WM surrounding the inferior and medial frontal gyrus, the basal ganglia and the somatosensory cortex (Bai et al., 2009). Thus, our result of WM alterations in schizophrenia patients with pronounced abnormal psychomotor behavior is in accordance with previous DTI studies and emphasizes the role of WM disconnectivity in motor pathways in disturbed motor behavior in schizophrenia (Walther, 2015).

Our results also confirm those of other DTI studies reporting associations between negative symptoms and WM microstructure in schizophrenia. Increased severity of negative symptoms measured by the Scale for the Assessment of Negative Symptoms (SANS) (Andreasen, 1989) was related to reduced WM integrity in the frontal lobe, corpus callosum, uncinate fasciculus, anterior thalamic radiation, inferior fronto-occipital fasciculus and superior longitudinal fasciculus (Asami et al., 2014, Nakamura et al., 2012, Szeszko et al., 2008, Zeng et al., 2016). Furthermore, WM microstructure in the splenium of the corpus callosum, the retrolenticular limb and posterior limb of the internal capsule have previously been associated with negative symptoms (Arnedo et al., 2015). Additionally, fibertracking studies revealed correlations of negative symptoms with WM connections between the ventral tegmental area and the amygdala, the nucleus accumbens and the orbitofrontal cortex and between the anterior cingulate cortex and the orbitofrontal cortex (Bracht et al., 2014, Ohtani et al., 2014, Ohtani et al., 2015). Contrary, there is one study of never-medicated first episode schizophrenia patients, which found no correlations between FA and negative symptoms (Cheung et al., 2011). However, this might be due to the fact that negative symptoms become more prominent at an advanced stage of the illness (Fenton and McGlashan, 1991). In line with the literature, we found reduced FA values in widespread WM regions of the frontal and temporal lobes in schizophrenia patients with prominent negative symptoms. This indicates that disruption of connectivity especially within tracts of the frontal lobe may contribute to the development of negative symptoms in schizophrenia (Khadka et al., 2013, Orliac et al., 2013, Venkataraman et al., 2012).

Some overlap was noted between the associations of FA and motor behavior and those of FA and negative symptoms, e.g. in the corpus callosum, corona radiata and the anterior limb of the internal capsule. This is not surprising as negative symptoms and motor symptoms often correlate at a descriptive level in schizophrenia (Walther and Strik, 2012), e.g. avolition contributes to reduced motor activity (Docx et al., 2013, Walther et al., 2015). In line with this, we found a strong correlation of the DSM-5 ratings for abnormal psychomotor behavior and negative symptoms in patients (see Table 2). Despite this behavioral overlap and shared associations of symptoms and WM microstructure in the anterior corona radiata and corpus callosum, there are clear differences between the WM associations of the two symptom dimensions: associations of FA with abnormal psychomotor behavior included right parietal and frontal clusters, whereas FA was associated in the posterior limb of internal capsule and temporal clusters with negative symptoms.

WM microstructure in the prefrontal and temporal lobe are often aberrant in schizophrenia (Federspiel et al., 2006, Kubicki et al., 2007). Our results are in line with these findings. In fact, we found reduced FA in the corpus callosum, corona radiata, internal capsule, inferior-fronto-occipital fasciculus and superior and inferior longitudinal fasciculus in patients compared to controls (see Supplementary Figure and Table). The clusters with group differences partially overlap with the associations between symptom ratings and WM microstructure. Therefore, a proportion of the associations with symptoms are located in areas with aberrant WM microstructure in schizophrenia. The inverse associations of FA and symptom severity indicate that patients with increased symptom severity in the investigated dimensions had the lowest FA values. This is in line with the group difference of reduced FA in patients. However, some of the detected clusters seem to be uniquely associated with symptom severity, such as bilateral external capsule, the posterior internal capsule and the uncinate fasciculus.

We found no significant associations between WM microstructure and the other three main symptom dimensions, delusions, hallucinations and disorganized speech. There are at least three reasons that might explain these findings. First, it is highly probable that the constructs behind these three DMS-5 dimension ratings are too complex to be mapped on brain structure because they include various underlying components. Second, the dimensions share less temporal stability compared to the motor and negative symptom dimensions. Therefore, a simple linear relationship with cerebral structure might not be an adequate way of investigating the neural basis of these dimensions, though functional correlates such as resting state perfusion proved suitable (Pinkham et al., 2015). Finally, the dimensions of abnormal psychomotor behavior and negative symptoms are not limited to the patients' symptom report but rely substantially on signs. Thus, the latter two symptom dimensions may have more validity.

In fact, previous literature provides very heterogeneous findings of WM correlates of positive symptoms in schizophrenia. Several studies found associations between delusions or hallucinations and WM microstructure in the arcuate fasciculus, cingulate bundle, corpus callosum, superior longitudinal fasciculus, inferior fronto-occipital fasciculus and the connection between the amygdala and the nucleus accumbens (Bracht et al., 2014, Hubl et al., 2004, Seok et al., 2007, Szeszko et al., 2008). In addition, recent studies found associations of hallucination and delusion severity with WM microstructure in the right inferior longitudinal fasciculus and the connection between the right posterior middle orbitofrontal cortex and the rostral part of the anterior cingulate cortex (Ohtani et al., 2015, Seitz et al., 2016). Only for delusions researchers found associations with WM properties in the cingulum bundle (Whitford et al., 2015), fornix and external capsule (Arnedo et al., 2015). Moreover, changes in WM microstructure of the left longitudinal fasciculus have recently been shown to correlate with improvements of positive symptoms (Zeng et al., 2016). However, there are other studies showing no associations of positive symptoms in schizophrenia and WM microstructure (Asami et al., 2014, Spalletta et al., 2013, Wolf et al., 2014). Regarding disorganized speech, only very few studies investigated the association with WM microstructure and found correlates in the middle and inferior longitudinal fasciculus (Asami et al., 2013, Phillips et al., 2009).

The observed heterogeneity of findings regarding WM and positive symptoms in the literature may originate from multiple factors, such as a wide range of methodological differences concerning the sample size, image analysis techniques, statistical techniques, subject characteristics and the heterogeneity of schizophrenia. More research is needed to disentangle the inconsistency of WM correlates of delusions and hallucinations and to confirm the preliminary results regarding disorganized speech.

Some limitations of the present study require discussion. First, some variables may influence WM microstructure. Almost all schizophrenia patients were treated with antipsychotic medication. Although previous studies found no effect of exposure to antipsychotic medication on WM structure (Kanaan et al., 2009, Peters et al., 2010) or WM disconnection in never medicated chronic schizophrenia patients (Liu et al., 2013), other studies show an effect of antipsychotic exposure on free radicals from activated microglia and on the release of inflammatory cytokines or lower astrocyte numbers in monkeys (Konopaske et al., 2008, Monji et al., 2009). Likewise, age may affect frontal and parietal WM structure (Davis et al., 2009, Gunning-Dixon et al., 2009, Salat et al., 2005). Age related WM changes occur in schizophrenia patients and healthy controls (HC), although in schizophrenia, FA impairments appear early in life and are permanently reduced compared to HC (Chiapponi et al., 2013). In addition, gender has previously been shown to affect WM microstructure in the corpus callosum, cingulum, cerebellar peduncle and superior longitudinal fasciculus (Kanaan et al., 2012, Kanaan et al., 2014, Menzler et al., 2011). Therefore, we entered chlorpromazine equivalents, age and gender as variables of no interest into all analyses. Second, the exact association between WM abnormalities and cross-sectional symptomatology bears some uncertainty. While WM alterations are considered relatively stable over time, symptom severity may wax and wane. Clearly, longitudinal studies in clinical populations may shed further light on this issue. However, given that WM microstructure contributes to functional connectivity of relevant brain areas, the association between brain structure and behavior is biologically plausible. This is particularly true for the motor dimension in which a defined cortico-subcortical loop is associated with the behavioral output (Walther, 2015).

In summary, the present study revealed characteristic patterns of WM alterations that were associated with the severity of abnormal psychomotor behavior and negative symptoms. These two DSM-5 symptom dimensions share brain-behavior associations and thus argue for pathobiological plausibility of the DSM-5 symptom dimensions in schizophrenia. Strikingly, relatively simple measures of symptom severity corroborated findings acquired with sophisticated instruments. Future studies could explore the longitudinal course of the symptomatology in schizophrenia and their white matter correlates.

## Conflict of interest

The authors have declared that there are no conflicts of interest in relation to the subject of this study.

## Funding source

This study received funding from the Bangerter-Rhyner Foundation (to SW) and the Swiss National Science Foundation (SNF grant 152619/1 to SW, AF and SB).

## Figures and Tables

**Table 1:** Demographic and clinical characteristics.

Variables	*M*	*SD*
Age (years)	37.20	10.40
Education (years)	13.53	3.10
Duration of illness (years)	11.77	11.05
Number of episodes	6.58	7.71
PANSS-Pos	18.25	6.48
PANSS-Neg	18.48	5.41
PANSS-Total	72.43	17.04
CPZ (mg)	411.70	358.47

PANSS, Positive and Negative Syndrome Scale; PANSS-Pos, subscale for positive symptoms; PANSS-Neg, subscale for negative symptoms; PANSS-Total, total score of PANSS; CPZ, chlorpromazine equivalents; *M* = Mean; *SD* = Standard deviation.

**Table 2:** Correlations between the DSM-5 symptom dimensions.

	Abnormal psychomotor behavior	Negative symptoms	Delusions	Hallucinations
Negative symptoms	0.509⁎⁎	–	–	–
Delusions	− 0.071	0.100	–	–
Hallucinations	0.205	0.342⁎	0.347⁎	–
Disorganized speech	− 0.064	− 0.121	0.107	0.071

Spearman's rho correlation coefficient.

⁎ Correlation is significant at the 0.05 level (2-tailed).

⁎⁎ Correlation is significant at the 0.01 level (2-tailed).

**Table 3:** Location of significant relationship between white matter microstructure (FA, RD) and abnormal psychomotor behavior.

Location	Center of gravity (mm coordinates)			Cluster size	*p* (*FWE corrected*)
x	y	z
*FA (negative linear relationship)*					
Genu of corpus callosum	0.20	27.71	6.86	817	0.039
Body of corpus callosum	− 3.07	10.05	26.02	530	0.040
Splenium of corpus callosum	19.49	− 42.08	26.76	119	0.049
Anterior limb of internal capsule R	19.15	9.30	9.24	348	0.038
Anterior corona radiata R	21.14	29.85	11.17	988	0.032
Anterior corona radiata L	− 18.32	31.24	10.76	593	0.042
Superior corona radiata R	24.91	1.66	28.29	302	0.034
Superior corona radiata L	− 17.62	8.96	34.84	117	0.042
Posterior thalamic radiation L	− 33.07	− 57.91	5.61	363	0.046
Sagittal stratum (include inferior longitudinal fasciculus and inferior fronto-occipital fasciculus) L	− 39.12	− 41.68	− 6.57	75	0.046
External capsule R	29.78	4.77	10.84	69	0.037
Anterior thalamic radiation R	19.53	19.21	7.80	322	0.035
Anterior thalamic radiation L	− 24.38	33.11	9.31	55	0.046
Forceps major	− 25.61	− 67.39	13.91	69	0.045
Forceps minor	− 1.24	36.55	6.67	1450	0.038
Inferior fronto-occipital fasciculus R	26.57	31.49	4.61	237	0.031
Inferior fronto-occipital fasciculus L	− 32.61	− 23.00	1.50	249	0.047
Inferior longitudinal fasciculus L	− 36.51	− 52.31	− 0.34	194	0.047
Superior longitudinal fasciculus R	35.85	− 21.92	32.11	566	0.036

*RD (positive linear relationship)*					
Genu of corpus callosum	2.05	29.60	10.74	200	0.049
Body of corpus callosum	− 1.27	13.82	25.77	239	0.047
Anterior limb of internal capsule R	20.31	10.45	10.85	75	0.050
Anterior corona radiata R	21.88	27.17	15.61	586	0.045
Anterior corona radiata L	− 17.43	27.87	21.17	167	0.049
Superior corona radiata R	21.06	10.62	31.27	89	0.044
Anterior thalamic radiation R	21.42	24.17	10.37	109	0.048
Forceps minor	6.96	36.70	11.12	445	0.047
Inferior fronto-occipital fasciculus R	26.48	31.79	4.65	151	0.049
Superior longitudinal fasciculus R	34.27	− 36.97	33.83	106	0.047

**Table 4:** Location of significant relationship between white matter microstructure (FA, RD) and negative symptoms.

Location	Center of gravity (mm coordinates)			Cluster size	*p* (*FWE corrected*)
x	y	z
*FA (negative linear relationship)*					
Genu of corpus callosum	1.07	28.34	8.24	1119	0.024
Body of corpus callosum	0.33	6.84	27.29	728	0.031
Cerebral peduncle R	17.41	− 17.46	− 8.22	155	0.043
Anterior limb of internal capsule R	18.40	8.11	8.42	530	0.024
Anterior limb of internal capsule L	− 17.55	12.05	6.09	331	0.033
Posterior limb of internal capsule R	21.58	− 11.40	7.74	360	0.032
Retrolenticular part of internal capsule R	32.07	− 27.06	5.23	308	0.029
Anterior corona radiata R	21.74	29.88	9.53	975	0.024
Anterior corona radiata L	− 19.31	30.78	9.29	906	0.025
Superior corona radiata R	24.08	− 2.38	26.93	283	0.033
Superior corona radiata L	− 17.53	8.51	35.17	122	0.025
Posterior thalamic radiation R	33.22	− 41.66	12.29	65	0.041
Sagittal stratum (include inferior longitudinal fasciculus and inferior fronto-occipital fasciculus) R	38.87	− 20.12	− 8.27	107	0.029
External capsule R	31.53	0.42	− 0.02	580	0.031
External capsule L	− 27.88	13.29	− 5.06	248	0.028
Fornix R	30.35	− 23.37	− 6.53	118	0.027
Anterior thalamic radiation R	18.17	14.22	6.81	449	0.025
Anterior thalamic radiation L	− 19.73	18.39	7.95	399	0.031
Corticospinal tract R	20.65	− 17.40	− 0.22	220	0.041
Forceps minor	0.78	36.96	9.31	2124	0.024
Inferior fronto-occipital fasciculus R	31.48	3.73	− 0.92	736	0.028
Inferior fronto-occipital fasciculus L	− 25.20	24.10	2.53	230	0.028
Uncinate fasciculus R	30.71	8.98	− 9.47	86	0.029
Uncinate fasciculus L	− 29.67	9.01	− 7.37	83	0.029

*RD (positive linear relationship)*					
Genu of corpus callosum	0.60	29.02	9.20	538	0.047
Body of corpus callosum	− 10.53	10.01	28.51	146	0.048
Anterior corona radiata R	18.61	32.52	14.48	338	0.045
Anterior corona radiata L	− 17.87	31.21	14.19	354	0.047
Superior corona radiata L	− 17.17	10.74	34.75	69	0.047
Forceps minor	2.44	38.75	9.59	1361	0.047

## Appendix A Supplementary data

Supplementary material. Image 1
