# Supplementary material for: Cerebral white matter structure is associated with DSM-5 schizophrenia symptom dimensions
Source: Neuroimage Clin. 2016 Jun 16;12:93–9. doi: 10.1016/j.nicl.2016.06.013 (PMC4925890; doi:10.1016/j.nicl.2016.06.013)
Supplement: Supplementary file 1 — Supplementary material. [file mmc1.docx]

Cerebral white matter structure is associated with DSM-5 schizophrenia symptom dimensions

Petra V. Viher, Katharina Stegmayer, Stéphanie Giezendanner, Andrea Federspiel, Stephan Bohlhalter, Tim Vanbellingen, Roland Wiest, Werner Strik, Sebastian Walther

**Supplement**

**Comparison of WM microstructure between schizophrenia patients and healthy controls**

Within a larger research project, we collected data from both schizophrenia patients and healthy control subjects. Forty-four (26 men, 18 women) controls were recruited among staff and students of the University Hospital of Psychiatry, Bern. Controls were matched for gender, age (*M* = 38.77 ±13.58) and education (*M* = 14.14 ±2.66). When compared to patients all *p*-values exceed 0.337 in the t-test on demographic parameters. The participants received oral and written information on the planned study and provided written informed consent.

Exclusion criteria were the same as in the patient sample. In addition, we excluded all control subjects with a history of any mental disorder or first-degree relatives with schizophrenia spectrum disorders.

We used the same methods regarding MRI acquisition, DTI processing and statistical analysis as in the patient sample. We entered age and gender as covariates of no interest into the analyses. The GLM examined a two-sample t-test of FA differences between patients and controls. We found significant reduction of FA in patients compared to controls in many different regions of the brain (*p* < 0.05, FWE corrected) (see Figure and Table).


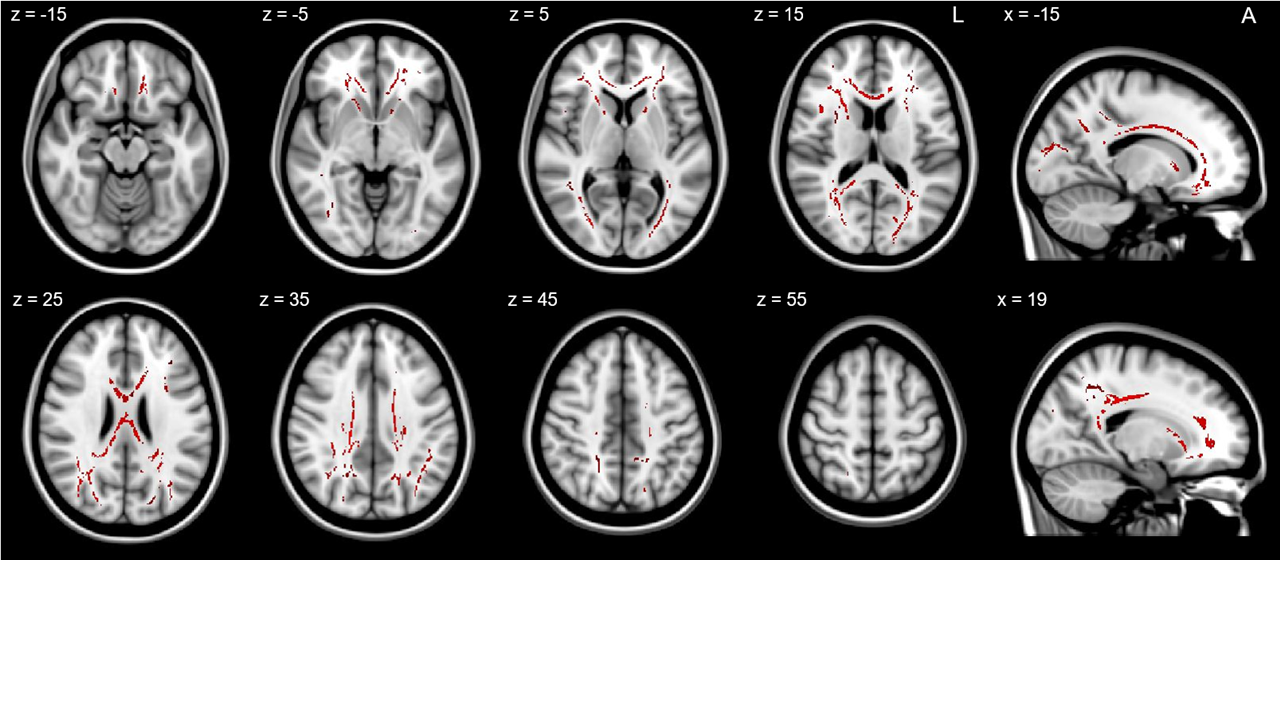


**Figure. Group differences of white matter microstructure between patients and controls.** The TBSS image shows the T-Test of controls > patients for FA values within the areas indicated in red at *p* < 0.05, FWE corrected. Z and x indicate the coordinates of the image slices in mm.

**Table. Group differences in white matter microstructure (FA) of controls > patients.**

| **Location** | **Center of Gravity (mm coordinates)** | | | **Cluster Size** | ***p* (FWE corrected)** |
| --- | --- | --- | --- | --- | --- |
|  | **x** | **y** | **z** |  |  |
| **FA** (negative linear relationship) |  |  |  |  |  |
| Genu of corpus callosum | 0.04 | 27.90 | 8.90 | 1027 | 0.018 |
| Body of corpus callosum | -0.10 | -7.45 | 28.53 | 1598 | 0.019 |
| Splenium of corpus callosum | 4.43 | -43.71 | 21.46 | 765 | 0.019 |
| Anterior limb of internal capsule R | 20.03 | 14.38 | 7.20 | 273 | 0.018 |
| Anterior limb of internal capsule L | -17.94 | 14.45 | 4.90 | 213 | 0.027 |
| Anterior corona radiata R | 21.29 | 31.09 | 5.20 | 704 | 0.020 |
| Anterior corona radiata L | -19.94 | 28.65 | 4.72 | 516 | 0.024 |
| Superior corona radiata R | 20.43 | -16.42 | 37.75 | 287 | 0.016 |
| Superior corona radiata L | -19.67 | -11.90 | 36.97 | 305 | 0.020 |
| Posterior corona radiata R | 23.47 | -41.51 | 30.88 | 251 | 0.018 |
| Posterior corona radiata L | -24.19 | -41.06 | 28.63 | 238 | 0.026 |
| Posterior thalamic radiation R | 32.38 | -60.74 | 8.23 | 459 | 0.023 |
| Posterior thalamic radiation L | -31.63 | -59.36 | 8.80 | 453 | 0.025 |
| Anterior thalamic radiation R | 20.61 | 17.90 | 7.87 | 268 | 0.019 |
| Anterior thalamic radiation L | -19.07 | 15.79 | 6.26 | 240 | 0.027 |
| Corticospinal tract R | 22.81 | -22.34 | 39.51 | 111 | 0.019 |
| Corticospinal tract L | -22.99 | -23.09 | 38.15 | 151 | 0.022 |
| Forceps major | 2.26 | -65.55 | 12.87 | 426 | 0.022 |
| Forceps minor | 1.80 | 32.64 | 5.72 | 1168 | 0.019 |
| Inferior fronto-occipital fasciculus R | 29.74 | -12.73 | 4.11 | 421 | 0.022 |
| Inferior fronto-occipital fasciculus L | -28.42 | -6.34 | 3.77 | 311 | 0.026 |
| Inferior longitudinal fasciculus R | 35.01 | -60.84 | -1.89 | 105 | 0.029 |
| Inferior longitudinal fasciculus L | -33.32 | -62.17 | 5.86 | 87 | 0.025 |
| Superior longitudinal fasciculus R | 38.31 | -42.34 | 19.00 | 179 | 0.034 |
| Superior longitudinal fasciculus L | -39.02 | -40.83 | 30.00 | 53 | 0.028 |
